# Potential for Mitochondrial DNA Sequencing in the Differential Diagnosis of Gynaecological Malignancies

**DOI:** 10.3390/ijms19072048

**Published:** 2018-07-13

**Authors:** Anna Myriam Perrone, Giulia Girolimetti, Martina Procaccini, Lorena Marchio, Alessandra Livi, Giulia Borghese, Anna Maria Porcelli, Pierandrea De Iaco, Giuseppe Gasparre

**Affiliations:** 1 Unit of Oncologic Gynecology, Sant Orsola-Malpighi Hospital, via Massarenti 13, 40138 Bologna, Italy; martina.procaccini@gmail.com (M.P.); alessandra.livi2@studio.unibo.it (A.L.); giuliamaria.borghese@gmail.com (G.B.); pierandrea.deiaco@unibo.it (P.D.I.); 2 Unit of Medical Genetics, Department of Medical and Surgical Sciences (DIMEC), Sant Orsola Hospital, Pav.11, via Massarenti 9, 40138 Bologna, Italy; giulsgiuls85@gmail.com (G.G.); lorenamarchio@gmail.com (L.M.); 3 Department of Pharmacy and Biotechnology (FABIT), University of Bologna, 40138 Bologna, Italy; annamaria.porcelli@unibo.it; 4 Center for Applied Biomedical Research (CRBA), University of Bologna, 40138 Bologna, Italy

**Keywords:** mitochondrial DNA, gynecological cancer, DNA genotyping, borderline ovarian tumors, synchronous tumors

## Abstract

In the event of multiple synchronous gynecological lesions, a fundamental piece of information to determine patient management, prognosis, and therapeutic regimen choice is whether the simultaneous malignancies arise independently or as a result of metastatic dissemination. An example of synchronous primary tumors of the female genital tract most frequently described are ovarian and endometrial cancers. Surgical findings and histopathological examination aimed at resolving this conundrum may be aided by molecular analyses, although they are too often inconclusive. High mitochondrial DNA (mtDNA) variability and its propensity to accumulate mutations has been proposed by our group as a tool to define clonality. We showed mtDNA sequencing to be informative in synchronous primary ovarian and endometrial cancer, detecting tumor-specific mutations in both lesions, ruling out independence of the two neoplasms, and indicating clonality. Furthermore, we tested this method in another frequent simultaneously detected gynecological lesion type, borderline ovarian cancer and their peritoneal implants, which may be monoclonal extra-ovarian metastases or polyclonal independent masses. The purpose of this review is to provide an update on the potential use of mtDNA sequencing in distinguishing independent and metastatic lesions in gynecological cancers, and to compare the efficiency of molecular analyses currently in use with this novel method.

## 1. The Issue of Synchrony in Gynecological Cancers

Tumors of the female genitalia are a heterogeneous group of disease with different prognosis even when originating from the same organ such as the uterus and ovary. A precise histological diagnosis of the tumor type is the cornerstone of targeted therapies in order to improve survival. Patient management becomes complicated when more than one active malignancy is diagnosed at the same time. Multiple synchronous gynecological lesions represent 1–2% of cases [1,2,3]: in this scenario, clinicians have to face the diagnostic problem of whether the simultaneous malignancies arise independently or as a result of metastatic dissemination [4,5]. This distinction is important especially with epithelial ovarian cancer, which accounts for the highest mortality among gynecological tumors [6,7].

The most frequently observed synchronous primary tumors of the female genital tract are ovarian cancer (OC) and endometrial cancer (EC) [8]. Simultaneous OC and EC could be diagnosed at their early stages and, generally, when presenting the following characteristics: (1) histological dissimilarity of the tumors (in tumor type, not grade, and with a cautionary note regarding the possibility of mixed tumors); (2) absent or merely superficial myometrial invasion of the endometrial tumor; (3) absence of vascular space invasion of EC; (4) additional presence of atypical endometrial hyperplasia; (5) absence of other evidences of spread of the EC; (6) ovarian unilateral tumor; (7) OC located in parenchyma; (8) no vascular space invasion, surface implants or predominant hilar location in the ovary; (9) absence of other evidences of spread of OC; (10) presence of ovarian endometriosis [9,10,11]. Nonetheless, some cases may not be classified with confidence, either because of widespread involvement, in which case the distinction is of academic rather than practical or prognostic significance, or, more importantly, because of overlapping or ambiguous histological features [3]. In these cases, a clear-cut diagnosis is difficult.

Borderline ovarian tumors (BOT) and peritoneal implants are another remarkable example of frequently observed multiple gynecological lesions. Invasive implants are associated with a lower survival rate [12], which makes it of paramount importance to understand whether a peritoneal implant is derived from BOT and therefore ought to be considered as metastasis, or whether it is an independent mass, i.e., a synchronous tumor to BOT.

In situations when two malignancies are diagnosed at the same time in a patient, clinicians must implement therapeutic regimens to cover both cancer types, at the same time trying to avoid negative impact on the overall outcome due to increased toxicity or relevant pharmacological interplay [13]. Because of the uniqueness of each tumor and of its intrinsic heterogeneity, distinguishing separate independent tumors from metastases remains extremely difficult. According to current guidelines, the diagnosis of the nature of simultaneously detected neoplasms is based on surgical findings and histopathological examination. Although molecular analyses may be additionally performed in order to aid diagnosis, these methods may be inconclusive and a relevant part of cases may not be diagnosed with confidence [14,15].

To fill the lack of a univocal method for diagnosis, different analyses have been proposed. Among these, mitochondrial DNA (mtDNA) sequencing has been recently brought to attention by our group to help define clonality in simultaneously detected gynecological tumors, aiding the identification of metastatic versus independent lesions in ambiguous interpretations where histopathological criteria and canonical molecular methods fail to be decisive [16]. The purpose of this review is to give an overview on the exploitation of mtDNA variability in gynecological malignancies in a critical comparison with currently implemented molecular analyses. mtDNA sequencing, as a method to detect synchronous lesions among gynecological tumors, has been indeed previously tested by our group to tackle the two previously mentioned clinical issues: to distinguish simultaneous versus metastatic EC/OC [17] and to demonstrate clonality of peritoneal implants of BOT [18].

## 2. Currently Used Molecular Analyses to Infer Tumor Clonality 

Patients diagnosed with multiple primary tumors or metastatic disease have a different prognosis and therapeutic choice, hence a careful discrimination is mandatory. Since it is not always possible to gain a clear understanding by using classical histopathological examination, the application of molecular and genetic techniques is often implemented. Different molecular analyses, including immunohistochemistry, DNA flow cytometry, loss of heterozygosity (LOH), gene mutation screening in *Phosphatase, tensin homologue/mutated in multiple advanced cancers (PTEN)/MMAC1*, *KRAS proto-oncogene (KRAS)*, *Tumour protein p53 (TP53)*, *Catenin, beta-1 (CTNNB1)*, X-Linked Clonality Analysis, alteration in β-catenin pathway, and microsatellite instability (MSI) have been adopted to aid diagnosis; however, to date there is no consensus on the most appropriate method [19,20,21,22,23,24,25]. In Table 1 we reported different methods used to identify synchronous versus metastatic tumors, their percentage of informativity as reported in the literature and the type of information that these methods can add to the diagnosis.

Previous studies suggest that the pattern of X-chromosome inactivation could predict whether two different tumors have a different origin. The advantage of this technique is that it can be applied to more than 90% of cases. In this context, it is possible to establish that two lesions are synchronous when cells show different patterns of X-chromosome inactivation. On the other hand, when cells share an identical patterns of X-chromosome inactivation, it is not possible to conclude that the two lesions are clonal, as in 50% of cases they may be synchronous primary carcinomas [19]. A further drawback of this method is that the detection of a minor monoclonal cell population within a background composed of non-tumor polyclonal cells is difficult and, in certain cases, monoclonal cells can reproduce the same pattern of X-chromosome inactivation of non-neoplastic cells [26].

Defects in DNA mismatch repair system is a common event in cancer and this may induce alterations in the size of microsatellite sequences during the replication of DNA in the tumor cells compared with the matched normal tissue.

Based on the presence of instability at specific loci in the two lesions, it is possible to infer either clonality or an independent nature of the two masses. Shenson et al. indicates MSI to be an informative method in the case of a lack of shared genetic alterations, implying independent developmental pathways [27], particularly for colorectal neoplasms with high MSI, which are known to be less prone to metastasis than microsatellite-stable tumors [28]. EC was reported with high incidence of MSI, whereas different studies have reported a very low frequency of MSI in OC [29,30,31,32]. Only one recent study has described a higher percentage of MSI in OC (Table 1), and the difference in efficiency was explained through the different methods used and the dependence of MSI frequency on histology of OC, being MSI less common in serous carcinoma [25]. The latter is the histotype found predominantly in stage III or IV, and usually giving rise to metastases. Based on these considerations, MSI analysis may not be highly informative for metastatic OC due to the very low frequency of MSI among series of synchronous tumors [31]. Furthermore, MSI also occurs by hypermethylation of the *MutL homolog 1 (MLH1)* promoter that is associated with the somatic *B-Raf Proto-Oncogene*, *Serine/Threonine Kinase (BRAF)* V600E mutation [33]. In tumors with frequent mutations of *BRAF* such as BOTs, this may not be considered as an independent tool.

Halperin et al. attempted to improve differential diagnosis using immunohistochemical (IHC) analysis, and reported that more than 60% of cases with synchronous tumors of the same histologic type could be differentiated from metastatic EC by distinct IHC expression of the estrogen and progesterone receptors [34].

A commonly used clonality marker is β-catenin localization. Generally, when the gene *CTNNB1* carries a missense mutation, β-catenin is localized in the nucleus, while stop codon mutations result in truncated protein products localized in the membrane. Nuclear immunoreactivity and *CTNNB1* mutations were associated with primary independent tumors, with favorable prognosis [22]. Nevertheless, an unequivocal diagnosis with this analysis is not feasible: β-catenin IHC analysis often shows a heterogeneous localization pattern, making the classification of tumors difficult. Moreover, it was already reported that independent primary tumors may display similar IHC profiles [3]. Localization of β-catenin is nonetheless related to mutation screening of *CTNNB1*, along with which may become a relatively reliable criterion. The weakness of *CTNNB1* mutations screening is that almost all mutations identified in human tumors are located in exon 3, making this a hot spot for mutation [35]. This is a common characteristic of oncogenes, which implies that only a few amino acids in the protein, most often only one, need to be mutated for its constitutive activation. Hence, the probability to find the same mutation in two independent tumors of the same patients by chance is higher in an oncogene than in a tumor suppressor gene that may be instead inactivated by a plethora of damaging mutations, especially if activation of that oncogene is required to drive tumorigenesis. Additionally, it was reported that identical gene mutations may be found in independent primaries due to a ‘field effect’ of a common oncogenic stimulus [22]. As a last further consideration, different abnormalities, such as subcellular localization or mutation occurrence, may be the effect of tumor heterogeneity rather than evidence of separate primaries [36].

Other oncogene/tumor suppressor gene mutations were used to distinguish primary tumors from metastasis such as *PTEN*, *MMAC1*, *KRAS*, *TP53*, *BRAF*. Tumors are generally characterized by genomic aberrations that include LOH, deletions, amplifications, and by an increased frequency of point mutations. Indeed, more than 50% patients with OC are found to harbor mutations in *TP53*, and 30–50% in *PTEN* [21,37]. Genomic aberrations may increase our understanding of tumor progression giving us information about clonal origin. Indeed, by comparing the genetic aberration profiles of two different lesions, it is feasible to infer clonal origin, whereby a mutation pattern may be considered as a genetic clonal marker, and different aberration profiles may indicate independent primary tumors [38,39]. Although genomic aberrations are a feature of nearly all human cancers and their accumulation takes place over time during tumor genesis and progression, mutations or LOH found in advanced-stage cancers may be absent from the early-stage mass, even if the lesions share the same clonal origin. In such cases, clonality may not easily be ruled out. Furthermore, the differences in deletions, point mutations and LOH pattern may be the result of tumor heterogeneity, making two clonal lesions different in their genetic profile [38,39]. Regarding point mutations, furthermore, common specific carcinogenic agents may induce similar genetic aberrations in two independent lesions [39].

Finally, in the last years an emerging field in diagnostics is the use of next-generation sequencing (NGS), such as whole-exome sequencing (WXS) and whole-genome sequencing (WGS). For synchronous gynecological tumors it was reported that NGS may be helpful to achieve a more accurate diagnosis and comprehensive characterization. In recent studies, using massive sequencing-based analyses (MPS) of synchronous EC and OC, the authors showed clonality despite development of a high genetic divergence after separation of the two neoplasms. In particular, Schultheis and coworkers targeted all exons and selected introns of 341 key cancer genes and, surprisingly, EC and OC in their sample set were shown to share similar somatic mutations and gene copy number alterations regardless of clinical classification [40,41,42]. Genomic studies focused on understanding the phylogenetic relationship of primary gynecological cancers and abdominopelvic metastases depict their clonal origins and progression patterns [43,44]. In a case report, WXS of tumor samples from a woman with well-differentiated endometrioid synchronous EC and OC revealed that the two carcinomas shared 57 nonsynonymous mutations [45]. In a study on bilateral OC, the authors showed with WXS and WGS that metastatic dissemination is often an early event, with dynamic mutational processes leading to divergent evolution and intratumor and intertumor heterogeneity [46]. These cases reveal the usefulness of NGS in defining the clonal relationships of synchronous tumors. Nevertheless, these techniques are based on mutation profiling and may suffer from the same issues reported for the oncogenes/oncosuppressor screening. 

The value of all these molecular features was explored by many authors although unequivocal data are still lacking [3]. Even if these techniques have proven useful in individual cases, their results should be interpreted with caution and always in concordance with clinico-pathologic findings [14].

## 3. Mitochondrial DNA Sequencing to Distinguish Simultaneous Versus Metastatic Gynecological Cancers

### 3.1. Advantages in the Use of Mitochondrial DNA Sequencing

**Figure 1:**
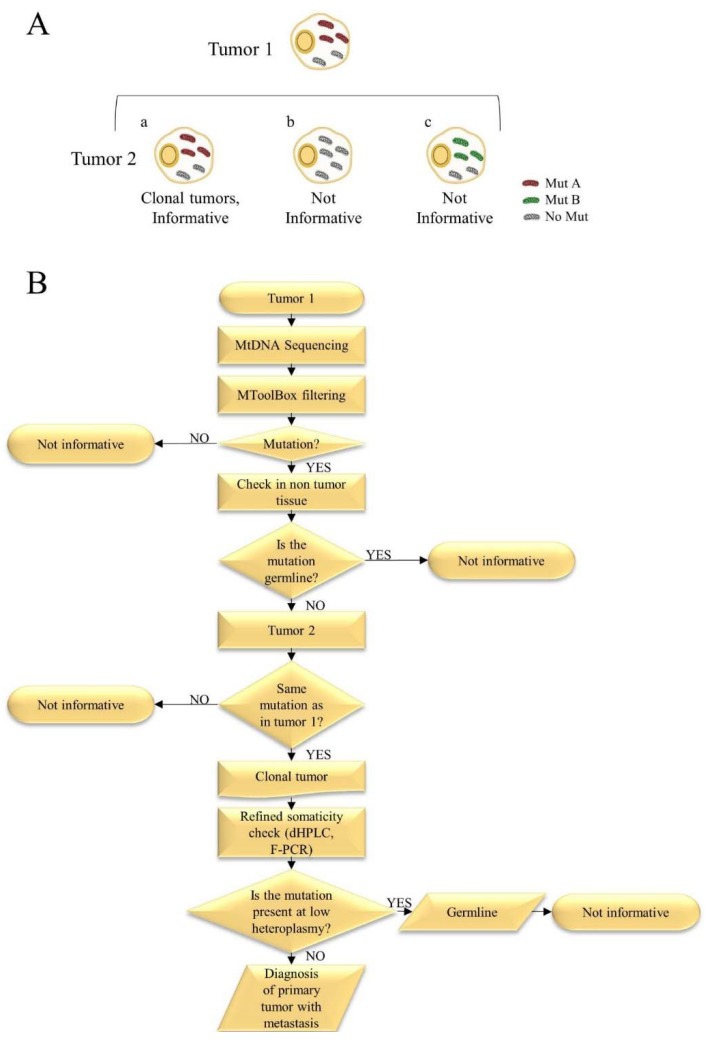
(**A**) Schematic representation of mtDNA informativity. Mitochondrial sequencing may be considered as informative only when a tumor-specific mutation is detected in both lesions of the same patient. All the mutations represented in the figure are non-germline mutations. In case (a), a tumor specific mutation occurs in both lesions, indicating clonality. In case (b), Tumor 1 carries a mutation that is not present in Tumor 2 making mtDNA sequencing not informative to infer clonality. In (c), Tumor 1 and 2 carry different mutations and mtDNA sequencing is not informative. In (b and c), it is not possible to rule out a clonal origin of the two masses. (**B**) The main steps of the mtDNA sequencing workflow: from DNA extraction to clonal diagnosis. mtDNA sequencing is easily and cheaply performed with Sanger method. Analysis of sequences is followed by MToolBox [93] filtering to select non-polymorphic variants. The latter are then checked in the non-tumor tissue or blood to ensure somaticity. If a tumor-specific mutation is detected in Tumor 1, the analysis is shifted to Tumor 2 to identify the same mutation as is Tumor 1. If Tumor 1 and Tumor 2 shared the same mutation a clonal origin may be hypothesized. A refined check of somaticity ought to be performed using more sensitive tools such as dHPLC and F-PCR [95] to rule out a germline mutation present at low heteroplasmic level in non-tumor tissue.

Mitochondria contain independent DNA that is circular, intron-free, and present at high copy numbers in all cells [50,51]. mtDNA is an often neglected small chromosome whose genetic peculiarities render it an exploitable instrument in the molecular biology of cancer. Among the most relevant features, mtDNA displays a high degree of variability which is deduced by the impressive and ever-growing number of fully sequenced human mtDNA genomes deposited in curated databases [52,53,54]. Moreover, polyplasmy implies that mtDNA is abundant in low cellularity samples, which allows for a feasible amplification and analysis even in small biopsies or fine-needle aspirates. A higher propensity of mtDNA to accumulate mutations than nuclear DNA is an additional hallmark that can be exploited for diagnostic purposes; this is due both to the heteroplasmic nature of mutations, that may coexist with wild-type mtDNA copies without triggering a phenotypic response (the so-called threshold effect), to the proximity of the mtDNA molecules to reactive oxygen species (ROS)-generating sites such as the respiratory complexes, accompanied by a lower capacity of DNA repair coupled with a high frequency of replicative errors. With all these characteristics, it is not surprising that cancer cells tend to harbor, at any time during cancer progression, different types and loads of mtDNA mutations [51], due to their highly proliferating rate as well as to the exposure to microenvironmental pressures such as hypoxia. Therefore, mtDNA mutations in cancer may be passenger events or become fixed when conferring an advantage, and act as modifiers of cancer progression [55]. Furthermore, in cancer tissues, no preferential hot spots have been identified among rRNA, tRNA, or coding genes and tumor-specific mutations are distributed uniformly across the mtDNA [56,57]. Deep sequencing has indeed shown that human cancer cells are rich with low loads of mtDNA mutations, most of which behave as functionally neutral, being at low heteroplasmy [51]. As a consequence, detection of a random somatic mtDNA mutation in two different neoplasms of the same patient may be considered as a marker of clonality of the two lesions, since it is reasonable to assume that two independent tumors arising in the same individual may not acquire the same somatic mtDNA genotype [58]. Interestingly, it has been already proposed that mtDNA may be used to trace lineages of epithelial cells [59], a rationale that prompted us to apply mtDNA sequencing to gynecological malignancies. Moreover, Arnold S et al. analyzed the mitochondrial genome of primary prostate cancer and two different metastases (soft tissue metastasis and bone metastasis) and normal tissue of 9 patients. Some tumor specific mutations were present in all three malignant tissues analyzed at different heteroplasmy levels. Other mutations were identified in the primary prostate and bone metastasis but did not appear in soft tissue metastasis and numerous mutations were found only in the bone but not in the primary or soft tissue metastasis [60]. Evidence is given, therefore, that during metastatization, cancer cells may acquire or lose mtDNA mutations according to their functional effects, random drift, or selective pressures. Based on this assumption, we are able to use mtDNA sequencing with the purpose of inferring clonality when the same mtDNA mutation is present in both tumor masses (Figure 1A).

### 3.2. Endometrial and Ovarian Cancers

OC and EC are the fifth and seventh most common neoplasms in women, respectively, and are characterized by a different prognosis, as 5-year survival for OC is less than 50% and for EC is much higher (75%) [61]. About 10% of women diagnosed with OC will be found to have synchronous EC, and in a mirrored fashion, about 5% of women with EC will be found to have simultaneous OC [3]. Synchronous tumors in the ovary and endometrium, when they occur, are the most frequent combination (50–70%) among all synchronous female genital tract malignancies. A precise diagnosis is hence mandatory, because it is related to a different prognosis and requires different surgical and post-operative management. 

Several studies reported that women with synchronous EC and OC have a better overall prognosis than patients with a single-organ cancer with ovarian or endometrial spread [62,63,64]. The median 5-year disease-free survival (DFS) rate has been found to be 65% for synchronous EC and OC, while is less than 50% for stage IIIA of EC with ovarian spread [65,66,67]. The correct recognition of the synchronous or metastatic nature of EC and OC is pivotal in choosing the most suitable therapeutic strategies and requires extreme caution. In particular, in the metastatic stages, with an overall survival less than 15% [5], external beam pelvic radiotherapy combined with chemotherapy and poly-chemotherapy are the appropriate adjuvant treatments for EC and OC, respectively [68,69]. A correct diagnosis of synchronous EC and OC or primary tumors with metastasis is important for both patient management and the scientific knowledge. Further, the elucidation of this issue is crucial because there are different opinions about the prognosis and treatment of these tumors related to primary or metastatic “*abitus*”. Some authors have reported a better overall prognosis for synchronous EC and OC compared to patients with a single-organ cancer with ovarian or endometrial spread [62,63,64], while others did not show a different outcome [70,71]. Early primary tumors of the uterus and the ovary have a good prognosis with overall survival for both sites between 80% and 90% and frequently these tumors do not require adjuvant therapies. Taking into account the risk of relapse and the good prognosis of EC and OC, chemotherapy with carboplatin and paclitaxel is useful even in EC and not concurrent treatments are required. Moreover, in synchronous EC and OC, radiation therapy is indicated for patients with advanced EC compounds, this latter treatment could be administered following chemotherapy [70].

According to current guidelines, the distinction between synchronous primary EC and OC and metastatic malignancy is based firstly on histopathological criteria, originally documented in 1985 and subsequently detailed in 1998 and in 2002 [9,10,11]. The application of common histopathological criteria poses significant problems both because it is an empiric tool based on the personal experience of the pathologist in charge and because it must take into account the heterogeneity of the samples analyzed. 

In some cases, histopathological examination is not adequate and molecular techniques may be fundamental to understand the nature of the two lesions. In our studies, we analyzed selected patients with a difficult diagnosis using the canonical molecular techniques compared with the use of mtDNA. First, in an attempt to assign a correct diagnosis in an isolated case of a woman with suspected synchronous OC and EC, we used mtDNA sequencing and results were validated through comparative genomic hybridization (aCGH), a technique that nevertheless is far more expensive and DNA-consuming than mtDNA sequencing, and whose introduction in cancer diagnostic routine may hardly be envisioned for the reasons discussed thus far [16].

Following that preliminary investigation, and with the aim to understand the potential of mtDNA as a tool to determine clonality, we applied mtDNA sequencing to assign a correct diagnosis in a cohort of synchronous OC and EC cases. We used a cheap and optimized Sanger technique to sequence the entire mtDNA [17] and compared mtDNA genotyping with currently used molecular techniques (MSI, β-catenin immunohistochemical staining/*CTNNB1* mutation screening) to show whether mtDNA sequencing may provide a more informative method in establishing diagnosis. We demonstrated that mtDNA sequencing was informative in nearly half of the cases, meaning that tumor-specific mutations were detected in both EC and OC of the same patient, ruling out independence of the two neoplasms and indicating clonality [72]. When additional somatic mutations are shown to occur in only one of the two tumors in our sample set, it is not possible to rule out a clonal origin of the two carcinomas, as these variants may indeed be subsequent to the initial clonal expansion and may only help reconstruct which one of the two is a metastasis (or a spread) of the other, if the germ-line sequence of the subject is available (Figure 1A). Based on this study [17], in ambiguous interpretations where histopathological criteria and canonical molecular methods fail to be conclusive, mtDNA analysis may help clinicians to formulate a diagnosis in about 45% of cases. Our results showed that mtDNA genotyping may provide a substantial contribution to recognize the metastatic nature of simultaneously detected EC and OC, helping in understanding the staging and clinical management of these patients. 

In our sample set [17], following diagnosis, all patients underwent surgical treatment followed by chemotherapy. To date, after at least 5 years from the diagnosis, 64% of the patients are free of disease. Apart from one old age patient (9%) who died for reasons not associated to the pathology, 3 patients (27%) presented relapses and one of them died from the disease. In our study, 2 of these patients were diagnosed with primary tumor of the ovary with metastasis in the endometrium. The last patient who showed relapse was diagnosed based on histopathological analysis with synchronous OC and EC but both ordinary molecular analysis and mtDNA analysis showed that the two tumors presented metastatic features in contrast to the histopathological diagnosis. It is reasonably to think that, according to molecular and mtDNA analyses, this case was represented by a primary tumor, probably of the ovary, with metastasis, highlighting the importance of molecular analyses for the correct diagnosis.

### 3.3. Borderline Ovarian Tumors (BOT) and Peritoneal Implants 

BOTs accounts for 10–20% of epithelial OCs and are characterized by histopathologic features and an intermediate behavior between clearly benign and frankly malignant OCs. Nearly 43–53% of BOTs have the serous histotype, while 42.5–52% are of mucinous type; the remaining 4–5% of BOTs are endometrioid, clear cell, mixed and transitional cell or Brenner type [72]. The standard guidelines for primary surgery in BOTs are similar to those for invasive OC, namely hysterectomy with bilateral salpingo-oophorectomy, multiple peritoneal biopsies, and peritoneal washing for cytology. In young patients, conservative fertility-sparing treatment is feasible and they may undergo surgery limited to a unilateral salpingo-oophorectomy with multiple peritoneal biopsies and washing for cytology [72,73]. Serous BOTs, the commonest histotype, may behave in an aggressive fashion with associated peritoneal lesions. Since women with extraovarian spread of disease have a very good prognosis, these peritoneal lesions are classified as implants instead of metastases [74]. Almost 35% of serous BOTs are associated with peritoneal implants, which may be classified as either invasive or noninvasive depending on their microscopic appearance: most implants are non-invasive, although invasive implants are found in 20–25% of cases [74]. Prognosis is generally excellent, patients without invasive implants have 10-year survival of 95%, which goes down to 60–70% in women with invasive peritoneal lesions [72]. However, after a follow-up of 15 years, 44% of patients diagnosed with serous BOTs with noninvasive implants presented recurrences and 25% died of disease [75]. A patient’s prognosis is mainly influenced by pathological stage, presence of post-operative residual disease, presence of peritoneal implants, and sub-classification of extra-ovarian disease as invasive or noninvasive. 

Because of their fundamental contribution to prognosis, several studies have attempted, with discordant results, to understand whether peritoneal implants originate from the spread of a single ovarian site or whether these nodules represent independent and polyclonal primary tumors [76,77]. Indeed, peritoneal implants are considered as independent masses of polyclonal origin by some authors, who underline their differences with the primary ovarian neoplasm as shown using X-chromosome inactivation and LOH pattern [78]. The hypothesis of a polyclonal origin of peritoneal implants and BOTs is in contrast to that brought forward for invasive epithelial OC, which has been demonstrated to be unifocal in origin and with a subsequent cancer cells spread in the peritoneal cavity [78,79,80,81]. The evidence of polyclonal origin of peritoneal implants may be consistent with the clinical observations on BOTs behavior, which may recur many years after initial surgical debulking suggesting that the relapse could be a new primary tumor due to the long time lap between the two tumors [59]. On the other hand, these differences between BOT and peritoneal implant may be the effect of the heterogeneity of the tumor during its spread in the peritoneal cavity. This hypothesis has been challenged by reports on clonality of BOTs and peritoneal implants demonstrated by studies on LOH profiling and the finding of identical *BRAF/KRAS* mutations in bilateral BOTs [76]. Nonetheless, mutation screening of *KRAS* and *BRAF* may be an only relatively reliable criterion to ascertain clonal origin, as codons 12 and 13 in *KRAS* and codon 600 in *BRAF* are some of the most commonly mutated regions identified in many human tumors, BOTs included [82], making these codons a mutational hot spot for *BRAF/KRAS* genes. Identical gene mutations may be therefore easily detected in independent lesions resulting from a common oncogenic stimulus in a frequently mutated gene region, and the finding of different genetic abnormalities may reflect tumor heterogeneity rather than evidence of separate primaries [36]. All these studies were not able to provide an undisputable proof to recognize the origin of the borderline tumor and the peritoneal lesions. However, the correct diagnosis of the nature of these lesions is mandatory for the evaluation of therapeutic options and for the prediction of prognosis. The concept of monoclonal origin of BOTs implies that the removal of the primary BOT should protect from the formation of implants, whereas a polyclonal origin would imply a “field effect”, i.e., that the whole peritoneal surface may give rise to BOTs implants. In the latter hypothesis, a surgical exploration of the entire peritoneal surface and multiple peritoneal biopsies should be mandatory, and radical surgery on reproductive organs such as contralateral ovary and uterus would therefore play a secondary role.

In this framework, mtDNA analysis was able to provide an indisputable proof to recognize the monoclonal origin of the BOT and the peritoneal lesions when the same tumor-specific mutation is found in both masses. For this reason, and since mtDNA sequencing had proven informative in detecting metastases of synchronous EC and OC, we implemented this technique in a pilot cohort of BOTs and their implants. Since these neoplasms are rare, we managed to collect 8 such cases for which DNA was available. Tumor-specific mtDNA mutations were detected in 62.5% of BOTs at different heteroplasmy levels. In nearly 40% of cases, the same mtDNA mutation was present in both BOT and the peritoneal implant revealing to be informative mutation that may be used to trace a clonal spread [18], strongly supporting the hypothesis previously brought forward that the origin of implants may be monoclonal, from the primary tumor. For these women, mtDNA analysis provided proof of the monoclonal origin of the BOT and the peritoneal lesion, representing the evidence that implants may arise as a consequence of a spread from a single ovarian site, based on mtDNA sequencing.

### 3.4. Other Applications of mtDNA Sequencing

In our experience, mtDNA sequencing has proven to be an invaluable tool in aiding diagnosis of clonality in simultaneous gynecological neoplasms, but the fields of application of this technique may be more extensive. Here, we have taken into consideration only synchronous gynecological malignancies with the higher incidence but other types of multiple gynecological tumors were reported with difficulties in diagnosis. For instance, most women diagnosed with epithelial OC display tumor spread in different sites of the peritoneal cavity and are treated as metastatic disease, although it was reported that individual foci could possibly originate from independent clones [83,84,85,86,87]. Moreover, in 20% t-25% of early-stage patients diagnosed with epithelial OC, without pelvic or metastasis extension, synchronous bilateral OC are reported [88]. In these cases, definition of clonality may have important implications for prognosis and patient management. Different studies supported a monoclonal origin of synchronous bilateral ovarian cancers [36,46,80,89]. Furthermore, different types of synchronous tumors other than OC and EC were reported in the peritoneal cavity, such as OC and EC each of which combined with cervix carcinoma. Some patients were reported with single genital tract malignancy coupled with primary extragenital malignancies [8]. In all these different situations in which the diagnosis of synchronous or metastatic cancer is difficult, the application of mtDNA sequencing may help in the classification of the tumors as metastatic neoplasia.

Another aspect to take into consideration is the co-occurrence of second primary malignancies in first cancer survivors, a growing population of ageing patients that is increasing [90]. Cancer survivors may develop second primary malignancies for different reasons such as cancer predisposition syndromes, environmental exposures, or late effects of therapies [91]. For these patients, it may be difficult to understand whether the second tumor is a different primary or a metastasis that may have developed from the initial cancer diagnosis [13]. In the case of a primary tumor with an informative mtDNA mutation, the second primary malignancy that carried the same mutation may be classified as metastases developed from the first cancer.

Last, we recently used mtDNA sequencing in a peculiar case of a woman with inguinal lymph nodes lesions as the first presentation of a focal intramucosal well differentiated endometrioid EC to provide an additional diagnostic confirmation of inguinal node metastasis from EC. Through mitochondrial genome sequencing it was possible to validate the histological examination and to understand whether the metastatic inguinal lymph nodes were derived from the EC, which demonstrated clonality of the two lesions [92].

## 4. Discussion and Conclusions

The issue of clonality in gynecological cancer has a high number of clinical implications. In the presence of simultaneous endometrial and ovarian neoplasia, their monoclonal origin implies a higher stage and calls for the use of more aggressive therapies, whereas a polyclonal origin is characterized by a good prognosis, requiring less drastic interventions. In the case of monoclonality of BOTs and their peritoneal implants, the removal of adnexa may prevent peritoneal spread, whereas polyclonality would imply a relapse risk from peritoneal surfaces. A correct diagnosis is therefore fundamental to determine prognosis. In this context, mtDNA analysis proved to be helpful in revealing clonality of two masses, the worst scenario for a patient, hence requiring aggressive intervention and treatment. Overall, only a subset of cases may be accurately categorized by conventional histopathological criteria and current diagnostic molecular techniques. A substantial fraction remains undetermined, calling for easy-to-perform and cheaper additional molecular tools that may solve ambiguities which particularly arise when molecular diagnosis is based on mutation of an oncogene. In this frame, mtDNA genotyping displays advantages when the same somatic mutation is found in both lesions.

Compared to mtDNA sequencing, other molecular methods such as MSI display lower informative power (Table 1), at least for gynecological malignancies where microsatellites are more stable than in other cancer types. β-catenin staining, on the other hand, does not appear to be too reliable as a method unless it is corroborated by a different molecular technique [17].

With respect to NGS-based techniques, these are nonetheless expensive, DNA- and time-consuming. Furthermore, the variants to take into consideration to infer clonality ought to be non-recurrent mutations and not oncogene mutations to avoid hotspots. In this framework, a large fraction of the human mtDNA variants reported in the reference human mitochondrial database HmtDB, included in the MSeqDR world-wide resource for mtDNA variants annotation [52,54], displays very low to zero variability. This occurs most frequently for somatic mutations, which makes it therefore reasonable to assume that two independent tumors diagnosed in a single patient may hardly acquire the same tumor-specific mtDNA mutations by chance. It is worth mentioning that recurrent cancer-associated mtDNA mutations have been reported at a relatively high frequency [51], and this ought to be taken into account when considering the informativity of mtDNA variants somatically found in multiple specimens. Indeed, the mutations that we reported in our previous studies displayed very low to zero variability values, rendering them useful to infer clonality, particularly when also the heteroplasmy level is comparable. Based on the different reliability of non-recurrent mtDNA mutations *versus* mutations in gene hotspots, it is possible to assume that also in the case of WXS or WGS, mutations in mtDNA may be used in combination with the mutation in genomic DNA to define clonality. The application of specific bioinformatics tools such as MToolBox [93], able to assemble mitochondrial genomes from WXS and/or WGS data, allows one to reconstruct and analyze human mtDNA from high-throughput sequencing and to find informative mtDNA mutations that may help, in correlation with nuclear variants, to formulate a proper diagnosis. Overall, although they are pivotal to understand the biology underlying cancer behavior, and for their use in research, it is hard to envision that WXS and WGS may immediately be implemented in diagnostic routine until the costs and time required for a proper data analysis are brought down.

In our sample set, using mtDNA sequencing, we have found a high rate of lesions, initially diagnosed as synchronous tumors, to be metastatic lesions. Our results are in line with results from other studies involving NGS and genetic analysis, that allows the identification of a high rate of metastatic lesions instead of synchronous EC and OC [94] supporting a recent paradigm-shifting concept that most diagnosed independent masses originate from a single tumor [17,40,41,94]. These recent findings highlighted the difficulties of a diagnosis based only on clinic-pathological criteria currently used to distinguish between dual primary tumors and single primary tumor with metastasis.

Additional advantages of mtDNA sequencing include a small amount of starting material, easy implementation and a standardized procedure that allows amplification by PCR with a single condition which may be implemented easily also on deep sequencing platforms, and the reliability of results. We have found that the informative power may be nearly 50%, accounting for a relevant number of ambiguous diagnoses that may therefore be resolved. In the remaining cases, mtDNA sequencing allows neither to infer clonality nor to rule it out. Nonetheless, this method, as others, is not drawback exempt, which derives from an intrinsic feature of mtDNA mutations, i.e., their heteroplasmic status. As we have previously shown, in order to be informative mtDNA mutations must be tumor-specific; this implies that whenever a mutation is detected, a careful check of somaticity ought to be performed to rule out the occurrence of the same mutation in non-tumor tissues, that may go unseen if Sanger sequencing is used for the analysis, as this method only allows for detection of about >10% heteroplasmy. Application of additional techniques such as liquid chromatography (dHPLC) or fluorescent PCR (F-PCR) [95] may be useful and cheap, but time-consuming and troublesome, although they represent the best standards, as we have shown when dealing with gynecological malignancies [18] (Figure 1B). This drawback, in our opinion, does not diminish the potential of mtDNA sequencing in aiding diagnosis, and will be likely overcome by the future implementation of NGS-based assays in diagnostic routines, specifically for such a small yet informative chromosome as mtDNA.

## Figures and Tables

**Table 1:** Comparison of the efficiency of different methods used to identify independent versus clonal gynecological tumors reported in the literature. The last column reports the type of diagnosis that the method may infer based on the literature. Abbreviations: IT: Independent Tumors; CT: Clonal Tumors, EC: Endometrial Cancer; OC: Ovarian Cancer; MSI: microsatellite instability; IHC: immunohistochemistry; LOH: loss of heterozygosity.

Technique	Efficiency	Type of Information
MSI	18% [17] 76% [25] 17–32% (EC) [30] 3–17% (OC) [30]	IT/CT
B-Catenin IHC	38% [47] 45.5% [17]	IT
*CTNNB1* mutation screening	44% [22] 25–38% [24]	IT/CT
Tumor suppressor mutation screening	30–50% [21] 88% [48]	IT/CT
X-chromosome inactivation	27% [25]	IT
LOH pattern	53% [5] 41% [49]	IT/CT
